# Supplementary material for: Nicotinic acetylcholine receptor modulator insecticides act on diverse receptor subtypes with distinct subunit compositions
Source: PLoS Genet. 2022 Jan 19;18(1):e1009920. doi: 10.1371/journal.pgen.1009920 (PMC8803171; doi:10.1371/journal.pgen.1009920)
Supplement: S1 Text — Fig A in S1 Text. The crossing schemes to establish the nAChRβ1R81T knock-in line. The HDR event was isolated by imidacloprid selection and confirmed by PCR. The vas-Cas9 (3XP3 RFP) was removed by the absence of red fluorescence in eyes. Fig B in S1 Text. Effects of nAChRβ1R81T point mutation on number of eggs laid (A), pupation rate of larvae (B) and negative geotaxis behavior (C). Fig C in S1 Text. Expression patterns of the nAChR genes in different KO mutants. Fig D in S1 Text. Phylogenetic relationships of core groups of nAChR subunits from 6 representative insect species including Apis mellifera (honey bee), Tribolium castaneum (red flour beetle), Myzus persicae (green peach aphid), Bombyx mori (silk worm), Bombus terrestris (bumble bee) and Drosophila melanogaster (fruit fly). The colorful dots at the nodes of the branches represent the values of bootstrap support for each branch. The D. melanogaster FMRFamide receptor (DmFR) was used as an outgroup. The sequence accession numbers are shown in Table N in S1 Text. Table A in S1 Text. Log dose probit mortality data and resistance ratios for imidacloprid. Table B in S1 Text. Log dose probit mortality data and resistance ratios for thiacloprid. Table C in S1 Text. Log dose probit mortality data and resistance ratios for acetamiprid. Table D in S1 Text. Log dose probit mortality data and resistance ratios for thiamethoxam. Table E in S1 Text. Log dose probit mortality data and resistance ratios for clothianidin. Table F in S1 Text. dose probit mortality data and resistance ratios for dinotefuran. Table G in S1 Text. Log dose probit mortality data and resistance ratios for nitenpyram. Table H in S1 Text. Log dose probit mortality data and resistance ratios for flupyradifurone. Table I in S1 Text. Log dose probit mortality data and resistance ratios for sulfoxaflor. Table J in S1 Text. Log dose probit mortality data and resistance ratios for triflumezopyrim. Table K in S1 Text. Log dose probit mortality data and resi [file pgen.1009920.s002.docx]

**S1 Text**

**Supporting Information for**

**Nicotinic acetylcholine receptor modulator insecticides act on diverse receptor subtypes with distinct subunit compositions**

Wanjun Lu, Zhihan Liu, Xinyu Fan, Xinzhong Zhang, Xiaomu Qiao and Jia Huang*

* Jia Huang

huangj[@zju.edu.cn](mailto:xmqiao@zju.edu.cn)

**Fig A in S1 Text.** The crossing schemes to establish the *nAChRβ1^R81T^* knock-in line. The HDR event was isolated by imidacloprid selection and confirmed by PCR. The *vas-Cas9* (3XP3 RFP) was removed by the absence of red fluorescence in eyes.

**Fig B in S1 Text.** Effects of *nAChRβ1^R81T^* point mutation on number of eggs laid (A), pupation rate of larvae (B) and negative geotaxis behavior (C).

**Fig C in S1 Text.** Expression patterns of the nAChR genes in different KO mutants.

**Fig D in S1 Text.** Phylogenetic relationships of core groups of nAChR subunits from 6 representative insect species including *Apis mellifera* (honey bee), *Tribolium castaneum* (red flour beetle), *Myzus persicae* (green peach aphid), *Bombyx mori* (silk worm), *Bombus terrestris* (bumble bee) and *Drosophila melanogaster* (fruit fly). The colorful dots at the nodes of the branches represent the values of bootstrap support for each branch. The *D. melanogaster* FMRFamide receptor (DmFR) was used as an outgroup. The sequence accession numbers are shown in Table N in S1 Text.

**Table A in S1 Text. Log dose probit mortality data and resistance ratios for imidacloprid**

**Table B in S1 Text. Log dose probit mortality data and resistance ratios for thiacloprid**

**Table C in S1 Text. Log dose probit mortality data and resistance ratios for acetamiprid**

**Table D in S1 Text. Log dose probit mortality data and resistance ratios for thiamethoxam**

**Table E in S1 Text. Log dose probit mortality data and resistance ratios for clothianidin**

**Table F in S1 Text. dose probit mortality data and resistance ratios for dinotefuran**

**Table G in S1 Text. Log dose probit mortality data and resistance ratios for nitenpyram**

**Table H in S1 Text. Log dose probit mortality data and resistance ratios for flupyradifurone**

**Table I in S1 Text. Log dose probit mortality data and resistance ratios for sulfoxaflor**

**Table J in S1 Text. Log dose probit mortality data and resistance ratios for triflumezopyrim**

**Table K in S1 Text. Log dose probit mortality data and resistance ratios for spinetoram**

**Table L in S1 Text. Sequence identities between *Drosophila* nAChR subunits and corresponding orthologs in other insects**

**Table M in S1 Text. Primers used in qPCR analysis**

**Table N in S1 Text. The accession numbers of sequences used in Fig D in S1 Text.**

G_0_ *_vas-Cas9_* _;_ $\frac{HDR}{+}$⮾

G_1_ $♂$*_vas-Cas9_*$;\frac{HDR}{+}or\frac{HDR}{HDR}or\frac{+}{+}$

Feed 96mg/L imidacloprid

G_1_ $♂\frac{HDR}{HDR}\times\frac{TM3, Sb}{TM6B, Tb}♀$

Select without fluorescence in eyes

Single fly PCR



G_2_$\frac{HDR}{TM6B, Tb}$⮾

G_3_$\frac{HDR}{HDR}$

**Fig A in S1 Text.** The crossing schemes to establish the *nAChRβ1^R81T^* knock-in line. The HDR event was isolated by imidacloprid selection and confirmed by PCR. The *vas-Cas9* (3XP3 RFP) was removed by the absence of red fluorescence in eyes.

**
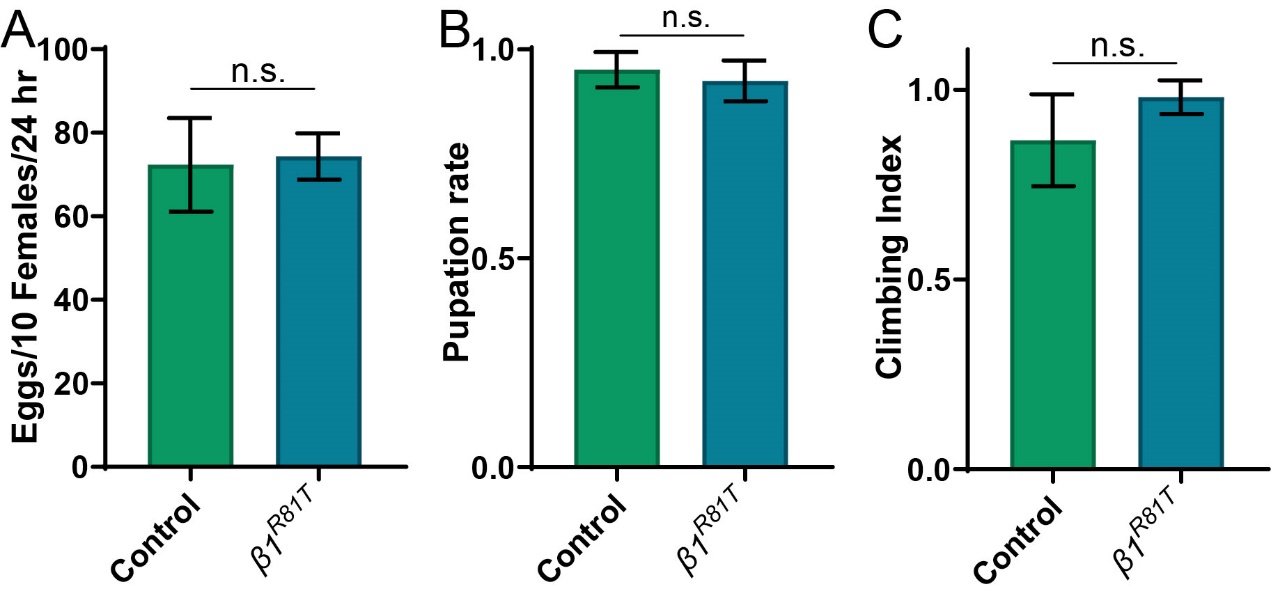
**

**Fig B in S1 Text.**  Effects of *nAChRβ1^R81T^* point mutation on number of eggs laid (A), pupation rate of larvae (B) and negative geotaxis behavior (C).

**
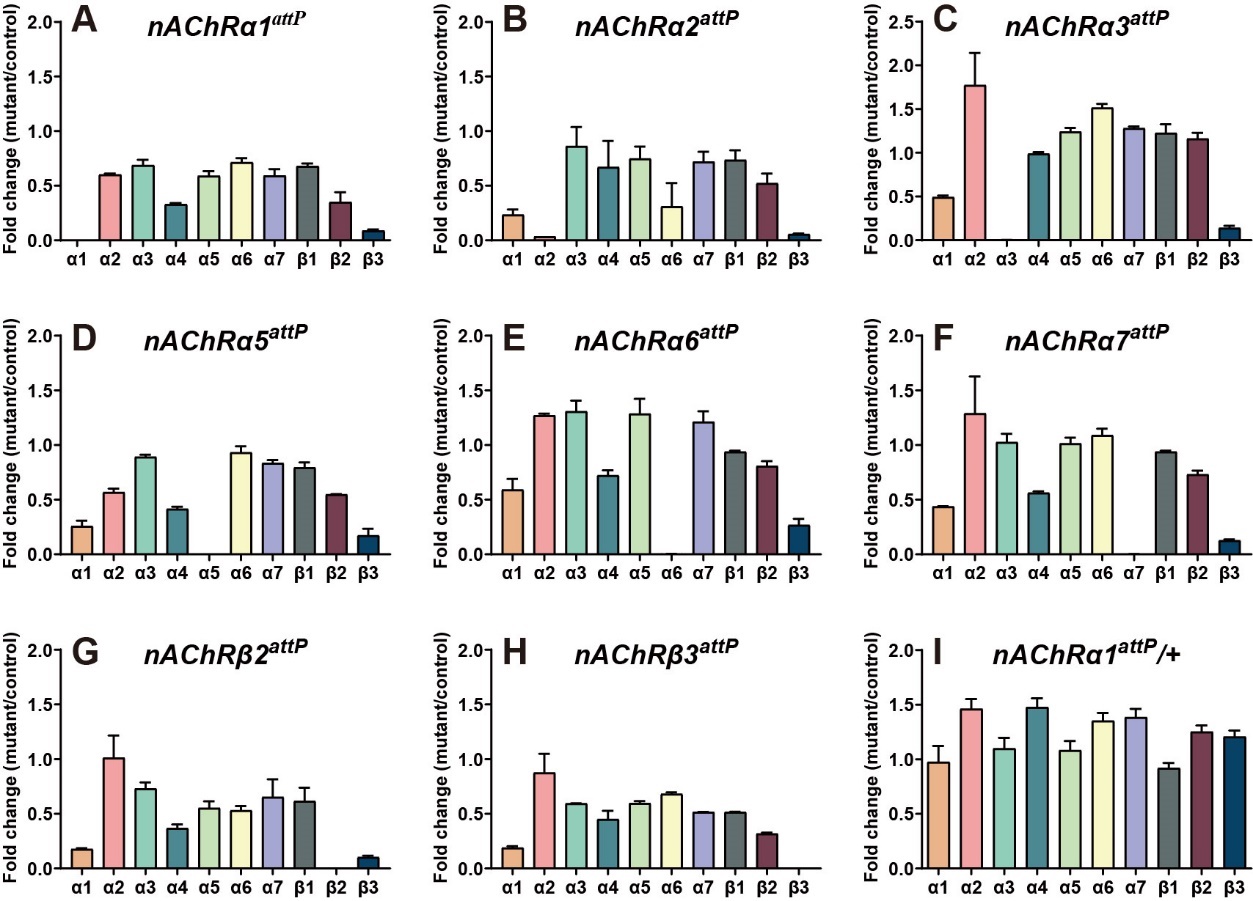
**

**Fig C in S1 Text.** Expression patterns of the nAChR genes in different KO mutants.


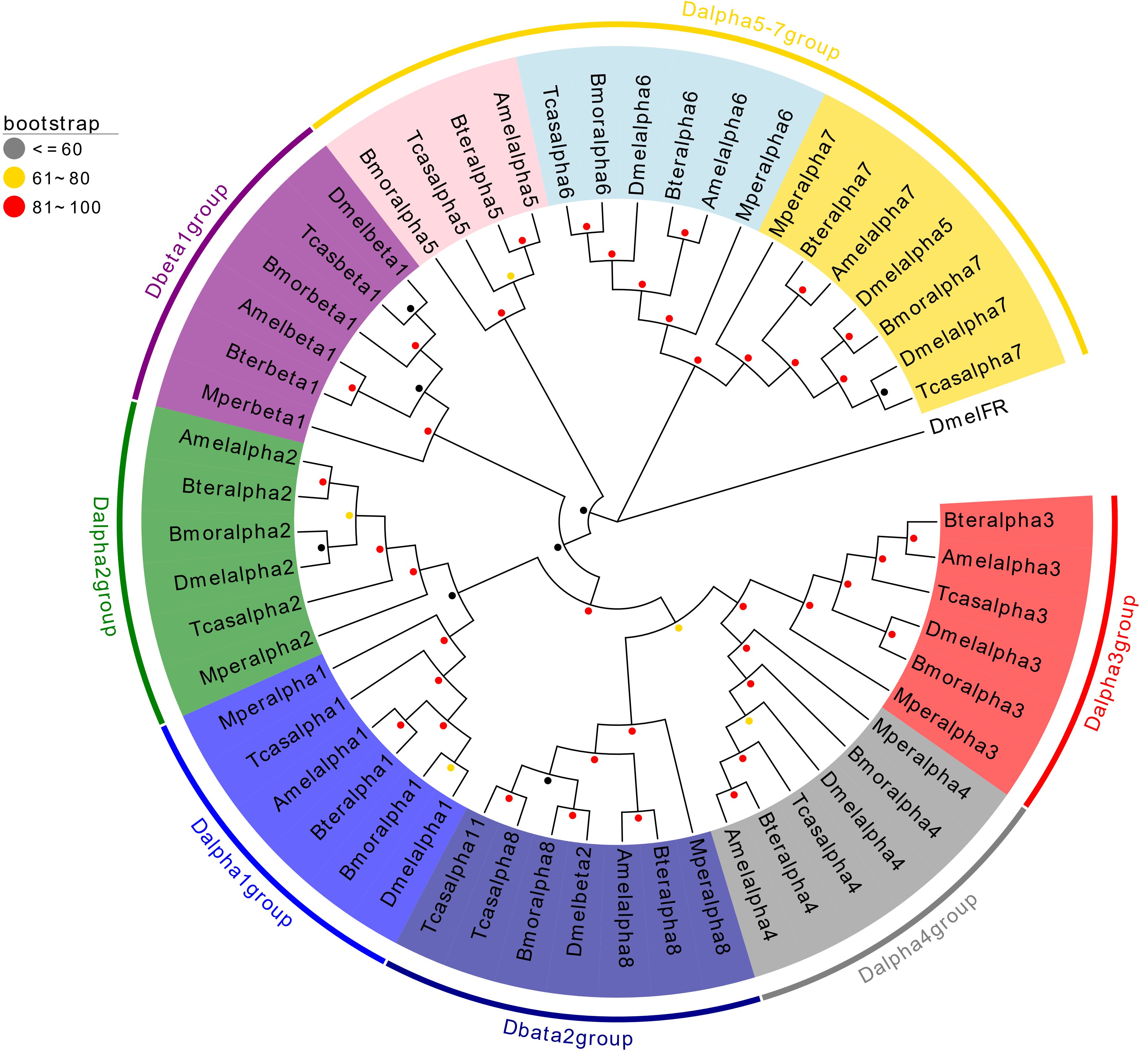


**Fig D in S1 Text.** Phylogenetic relationships of core groups of nAChR subunits from 6 representative insect species including *Apis mellifera* (honey bee), *Tribolium castaneum* (red flour beetle), *Myzus persicae* (green peach aphid), *Bombyx mori* (silk worm), *Bombus terrestris* (bumble bee) and *Drosophila melanogaster* (fruit fly). The colorful dots at the nodes of the branches represent the values of bootstrap support for each branch. The *D. melanogaster* FMRFamide receptor (DmFR) was used as an outgroup. The sequence accession numbers are shown in **Table N in S1 Text.**

**Table A in S1 Text. Log dose probit mortality data and resistance ratios for imidacloprid**

| **Strain** | **LC_50_**  **(mg/L)** | **95% CL** | **LC_95_**  **(mg/L)** | **95% CL** | **Resistance ratio** | |
| --- | --- | --- | --- | --- | --- | --- |
|  |  |  |  |  | **LC_50_** | **LC_95_** |
| Control | 8.6 | 7.5-9.9 | 26.6 | 21.5-35.9 | 1.0 | 1.0 |
| *α1^-/-^* | 296.7 | 161.4-519.4 | 14555 | 5396.3-85114.0 | 34.5 | 547.2 |
| *α1^+/-^* | 150.4 | 74.7-272.7 | 935.5 | 456.0-5243.1 | 17.5 | 35.2 |
| *α2^-/-^* | 417.0 | 254.8-686.0 | 3113.5 | 1549.4-13500.0 | 48.5 | 117.0 |
| *α2^+/-^* | 52.5 | 38.7-69.6 | 114.0 | 82.2-259.1 | 6.1 | 4.3 |
| *α3^-/-^* | 10.7 | 9.7-11.7 | 16.7 | 14.7-21.1 | 1.2 | 0.6 |
| *α4^T227M^* | 22.9 | 19.2-27.2 | 58.0 | 45.2-86.9 | 2.7 | 2.2 |
| *α4^T227M^/+* | 24.1 | 16.9-35.5 | 53.5 | 36.1-178.2 | 2.8 | 2.0 |
| *α5^-/-^* | 9.6 | 7.5-12.1 | 27.5 | 19.9-50.2 | 1.1 | 1.0 |
| *α6^-/-^* | 7.8 | 6.2-10.0 | 28.2 | 19.9-49.7 | 0.9 | 1.1 |
| *α7^-/-^* | 22.8 | 20.4-25.5 | 43.6 | 37.1-55.8 | 2.7 | 1.6 |
| *β1^R81T^* | 565.7 | 407.3-781.4 | 2885.7 | 1835.2-6036.1 | 65.8 | 108.5 |
| *β1^R81T^/+* | 87.3 | 73.8-103.4 | 210.3 | 165.0-312.2 | 10.2 | 7.9 |
| *β2^-/-^* | 725.4 | 513.6-1027.5 | 4702.2 | 2840.2-10564.0 | 84.3 | 176.8 |
| *β2^+/-^* | 24.7 | 21.6-28.4 | 42.3 | 35.1-60.5 | 2.9 | 1.6 |
| *β3^-/-^* | 13.1 | 11.6-14.7 | 27.5 | 23.1-35.6 | 1.5 | 1.0 |

**Table B in S1 Text. Log dose probit mortality data and resistance ratios for thiacloprid**

| **Strain** | **LC_50_**  **(mg/L)** | **95% CL** | **LC_95_**  **(mg/L)** | **95% CL** | **Resistance ratio** | |
| --- | --- | --- | --- | --- | --- | --- |
|  |  |  |  |  | **LC_50_** | **LC_95_** |
| Control | 30.4 | 28.3-32.6 | 49.3 | 44.6-56.7 | 1.0 | 1.0 |
| *α1^-/-^* | 2674.9 | 2007.8-3553.3 | 8940.1 | 6082.5-17994.0 | 88.0 | 181.3 |
| *α1^+/-^* | 89.2 | 67.1-116.2 | 182.2 | 134.3-394.2 | 2.9 | 3.7 |
| *α2^-/-^* | 1031.0 | 783.7-1384.3 | 14812.0 | 8693.3-31445.0 | 33.9 | 300.4 |
| *α2^+/-^* | 37.3 | 31.6-43.9 | 86.2 | 68.2-126.3 | 1.2 | 1.7 |
| *α3^-/-^* | 36.9 | 31.0-43.8 | 93.4 | 73.1-138.0 | 1.2 | 1.9 |
| *α4^T227M^* | 27.0 | 16.7-40.5 | 106.2 | 63.4-357.9 | 0.9 | 2.2 |
| *α5^-/-^* | 20.0 | 16.9-23.6 | 46.7 | 36.9-68.4 | 0.7 | 0.9 |
| *α6^-/-^* | 26.1 | 22.6-29.9 | 78.5 | 63.8-105.1 | 0.9 | 1.6 |
| *α7^-/-^* | 40.2 | 33.7-47.5 | 95.3 | 75.1-142.7 | 1.3 | 1.9 |
| *β1^R81T^* | 1342.7 | 802.5-2229.7 | 8533.6 | 4418.4-32285.0 | 44.2 | 173.1 |
| *β1^R81T^/+* | 40.3 | 26.3-62.2 | 106.9 | 67.4-458.1 | 1.3 | 2.2 |
| *β2^-/-^* | 1677.8 | 922.8-3425.5 | 21015.0 | 8759.4-98473.0 | 54.3 | 401.8 |
| *β2^+/-^* | 11.8 | 8.7-14.4 | 32.5 | 24.6-58.3 | 0.4 | 0.7 |
| *β3^-/-^* | 39.4 | 33.5-46.4 | 88.0 | 70.0-128.4 | 1.3 | 1.8 |

**Table C in S1 Text. Log dose probit mortality data and resistance ratios for acetamiprid**

| **Strain** | **LC_50_**  **(mg/L)** | **95% CL** | **LC_95_**  **(mg/L)** | **95% CL** | **Resistance ratio** | |
| --- | --- | --- | --- | --- | --- | --- |
|  |  |  |  |  | **LC_50_** | **LC_95_** |
| Control | 9.7 | 7.4-13.1 | 22.0 | 15.5-50.7 | 1.0 | 1.0 |
| *α1^-/-^* | 131.3 | 118.0-146.2 | 239.4 | 204.7-306.1 | 13.5 | 10.9 |
| *α1^+/-^* | 43.6 | 31.0-59.2 | 90.2 | 64.7-240.8 | 4.5 | 4.1 |
| *α2^-/-^* | 15.2 | 11.4-20.5 | 37.9 | 26.2-89.1 | 1.6 | 1.7 |
| *α3^-/-^* | 16.9 | 14.7-19.5 | 43.1 | 34.5-61.8 | 1.7 | 2.0 |
| *α4^T227M^* | 9.4 | 8.2-10.9 | 17.3 | 14.2-24.8 | 1.0 | 0.8 |
| *α5^-/-^* | 10.1 | 8.8-11.5 | 23.9 | 19.7-32.0 | 1.0 | 1.1 |
| *α6^-/-^* | 22.6 | 18.5-28.0 | 63.7 | 46.6-109.0 | 2.3 | 2.9 |
| *α7^-/-^* | 27.4 | 18.8-41.2 | 55.3 | 38.0-204.6 | 2.8 | 2.5 |
| *β1^R81T^* | 231.5 | 214.7-249.5 | 397.7 | 355.5-465.5 | 23.9 | 18.1 |
| *β1^R81T^/+* | 31.2 | 27.4-35.4 | 50.5 | 43.0-67.6 | 3.2 | 2.3 |
| *β2^-/-^* | 126.5 | 109.6-146.4 | 238.3 | 195.2-340.1 | 13.0 | 10.8 |
| *β2^+/-^* | 24.9 | 22.1-28.3 | 38.0 | 32.4-51.8 | 2.6 | 1.7 |
| *β3^-/-^* | 17.7 | 13.5-23.3 | 55.6 | 38.2-111.7 | 1.8 | 2.5 |

**Table D in S1 Text. Log dose probit mortality data and resistance ratios for thiamethoxam**

| **Strain** | **LC_50_(mg/L)** | **95% CL** | **LC_95_(mg/L)** | **95% CL** | **Resistance ratio** | |
| --- | --- | --- | --- | --- | --- | --- |
|  |  |  |  |  | **LC_50_** | **LC_95_** |
| Control | 8.2 | 7.6-8.9 | 15.3 | 13.4-18.4 | 1.0 | 1.0 |
| *α1^-/-^* | 30.5 | 22.7-41.7 | 65.8 | 46.6-158.5 | 3.7 | 4.3 |
| *α2^-/-^* | 7.9 | 6.8-9.2 | 14.9 | 12.2-21.2 | 1.0 | 1.0 |
| *α3^-/-^* | 32.9 | 29.5-36.4 | 71.2 | 61.6-87.1 | 4.0 | 4.7 |
| *α4^T227M^* | 4.1 | 2.6-5.9 | 9.9 | 6.6-34.8 | 0.5 | 0.6 |
| *α5^-/-^* | 7.3 | 6.6-8.0 | 11.3 | 10.0-13.6 | 0.9 | 0.7 |
| *α6^-/-^* | 10.8 | 10.0-11.6 | 16.7 | 14.8-20.4 | 1.3 | 1.1 |
| *α7^-/-^* | 8.2 | 7.2-9.3 | 16.5 | 13.7-22.1 | 1.0 | 1.1 |
| *β1^R81T^* | 1935.5 | 1756.0-2142.6 | 4783.9 | 4047.9-5980.8 | 236.0 | 312.7 |
| *β1^R81T^/+* | 18.3 | 12.9-25.0 | 38.4 | 27.4-97.2 | 2.2 | 2.5 |
| *β2^-/-^* | 12.8 | 11.0-14.9 | 25.8 | 20.9-37.0 | 1.6 | 1.7 |
| *β3^-/-^* | 11.8 | 8.9-20.0 | 22.6 | 15.4-121.0 | 1.4 | 1.5 |

**Table E in S1 Text. Log dose probit mortality data and resistance ratios for clothianidin**

| **Strain** | **LC_50_**  **(mg/L)** | **95% CL** | **LC_95_**  **(mg/L)** | **95% CL** | **Resistance ratio** | |
| --- | --- | --- | --- | --- | --- | --- |
|  |  |  |  |  | **LC_50_** | **LC_95_** |
| Control | 3.5 | 3.1-3.9 | 8.4 | 7.0-10.9 | 1.0 | 1.0 |
| *α1^-/-^* | 10.3 | 9.2-11.4 | 20.5 | 17.5-26.1 | 2.9 | 2.4 |
| *α2^-/-^* | 4.7 | 4.3-5.2 | 8.5 | 7.4-10.5 | 1.3 | 1.0 |
| *α3^-/-^* | 11.5 | 8.1-16.7 | 25.9 | 17.5-78.1 | 3.3 | 3.1 |
| *α4^T227M^* | 2.5 | 2.1-2.9 | 6.3 | 4.9-9.5 | 0.7 | 0.8 |
| *α5^-/-^* | 2.8 | 2.5-3.2 | 4.4 | 3.8-5.7 | 0.8 | 0.5 |
| *α6^-/-^* | 5.1 | 4.6-5.7 | 9.4 | 8.1-11.9 | 1.5 | 1.1 |
| *α7^-/-^* | 6.1 | 5.3-6.9 | 17.3 | 14.1-23.2 | 1.7 | 2.1 |
| *β1^R81T^* | 969.4 | 730.6-1278.8 | 4940.0 | 3300.4-9182.5 | 277.0 | 588.1 |
| *β1^R81T^/+* | 13.4 | 11.8-15.2 | 21.2 | 18.1-28.7 | 3.8 | 2.5 |
| *β2^-/-^* | 3.4 | 3.0-4.0 | 6.2 | 5.2-8.6 | 1.0 | 0.7 |
| *β3^-/-^* | 7.6 | 7.0-8.4 | 13.5 | 11.7-16.9 | 2.2 | 1.6 |

**Table F in S1 Text. Log dose probit mortality data and resistance ratios for dinotefuran**

| **Strain** | **LC_50_**  **(mg/L)** | **95% CL** | **LC_95_**  **(mg/L)** | **95% CL** | **Resistance ratio** | |
| --- | --- | --- | --- | --- | --- | --- |
|  |  |  |  |  | **LC_50_** | **LC_95_** |
| Control | 5.5 | 5.2-5.9 | 11.4 | 10.2-13.0 | 1.0 | 1.0 |
| *α1^-/-^* | 15.0 | 13.6-17.0 | 25.9 | 21.7-34.8 | 2.7 | 2.3 |
| *α2^-/-^* | 7.2 | 6.5-7.9 | 12.3 | 10.7-15.4 | 1.3 | 1.1 |
| *α3^-/-^* | 30.4 | 27.6-33.2 | 55.0 | 48.4-66.0 | 5.5 | 4.8 |
| *α3^+/-^* | 16.2 | 14.1-18.7 | 29.2 | 24.2-41.3 | 2.9 | 2.6 |
| *α4^T227M^* | 3.7 | 3.2-4.3 | 7.0 | 5.7-10.2 | 0.7 | 0.6 |
| *α5^-/-^* | 6.5 | 6.0-7.2 | 9.7 | 8.6-11.8 | 1.2 | 0.9 |
| *α6^-/-^* | 8.7 | 7.8.-9.8 | 16.7 | 14.1-21.7 | 1.6 | 1.5 |
| *α7^-/-^* | 7.3 | 6.7-8.0 | 10.5 | 9.5-12.2 | 1.3 | 0.9 |
| *β1^R81T^* | 2190.9 | 1790.6-2702.1 | 4803.3 | 3659.6-7985.8 | 398.3 | 421.3 |
| *β1^R81T^/+* | 9.3 | 8.1-10.6 | 15.6 | 13.0-22.2 | 1.7 | 1.4 |
| *β2^-/-^* | 10.3 | 9.0-11.8 | 17.6 | 14.7-24.7 | 1.9 | 1.5 |
| *β3^-/-^* | 11.4 | 8.0-20.6 | 23.7 | 15.4-201.0 | 2.1 | 2.1 |

| **Strain** | **LC_50_**  **(mg/L)** | **95% CL** | **LC_95_**  **(mg/L)** | **95% CL** | **Resistance ratio** | |
| --- | --- | --- | --- | --- | --- | --- |
|  |  |  |  |  | **LC_50_** | **LC_95_** |
| Control | 19.8 | 17.9-22.2 | 36.6 | 30.8-48.9 | 1.0 | 1.0 |
| *α1^-/-^* | 54.4 | 43.5-69.5 | 115.2 | 85.4-220.8 | 2.7 | 3.1 |
| *α2^-/-^* | 15.1 | 13.4-17.0 | 33.1 | 27.7-43.0 | 0.8 | 0.9 |
| *α3^-/-^* | 53.7 | 44.8-64.3 | 147.3 | 113.2-223.5 | 2.7 | 4.0 |
| *α4^T227M^* | 13.4 | 11.2-15.7 | 28.9 | 23.0-44.3 | 0.7 | 0.8 |
| *α5^-/-^* | 16.0 | 14.5-17.5 | 24.7 | 22.0-29.9 | 0.8 | 0.7 |
| *α6^-/-^* | 19.4 | 16.1-24.8 | 35.4 | 27.7-61.1 | 1.0 | 1.0 |
| *α7^-/-^* | 13.6 | 12.2-15.2 | 25.9 | 22.0-33.2 | 0.7 | 0.7 |
| *β1^R81T^* | 3629.0 | 2906.8-4770.6 | 11394.0 | 7852.2-21070.0 | 183.3 | 311.3 |
| *β1^R81T^/+* | 30.3 | 26.6-34.7 | 51.4 | 43.2-70.4 | 1.5 | 1.4 |
| *β2^-/-^* | 29.6 | 25.8-34.0 | 52.2 | 43.4-72.6 | 1.5 | 1.4 |
| *β3^-/-^* | 17.1 | 15.5-19.0 | 29.6 | 25.5-37.5 | 0.9 | 0.8 |

**Table G in S1 Text. Log dose probit mortality data and resistance ratios for nitenpyram**

| **Strain** | **LC_50_**  **(mg/L)** | **95% CL** | **LC_95_**  **(mg/L)** | **95% CL** | **Resistance ratio** | |
| --- | --- | --- | --- | --- | --- | --- |
|  |  |  |  |  | **LC_50_** | **LC_95_** |
| Control | 38.8 | 36.1-41.8 | 74.6 | 66.3-87.5 | 1.0 | 1.0 |
| *α1^-/-^* | 59.2 | 52.7-66.6 | 121.6 | 102.2-157.6 | 1.5 | 1.6 |
| *α2^-/-^* | 53.6 | 49.0-59.7 | 100.1 | 85.4-127.9 | 1.4 | 1.3 |
| *α3^-/-^* | 108.1 | 98.4-119.0 | 171.0 | 150.4-208.7 | 2.8 | 2.3 |
| *α4^T227M^* | 33.0 | 28.5-38.3 | 64.3 | 52.3-92.5 | 0.9 | 0.9 |
| *α5^-/-^* | 34.9 | 26.8-46.2 | 88.8 | 62.3-187.3 | 0.9 | 1.2 |
| *α6^-/-^* | 55.3 | 33.2-99.2 | 129.7 | 79.8-1051.9 | 1.4 | 1.7 |
| *α7^-/-^* | 62.5 | 57.1-68.5 | 127.0 | 110.4-153.7 | 1.6 | 1.7 |
| *β1^R81T^* | 842.0 | 767.5-924.5 | 2151.2 | 1849.8-2616.5 | 21.7 | 28.8 |
| *β1^R81T^/+* | 81.6 | 70.7-94.4 | 151.8 | 124.8-214.9 | 2.1 | 2.0 |
| *β2^-/-^* | 60.7 | 53.1-69.3 | 102.9 | 86.5-140.9 | 1.6 | 1.4 |
| *β3^-/-^* | 54.5 | 46.2-64.5 | 91.2 | 74.5-137.6 | 1.4 | 1.2 |

**Table H in S1 Text. Log dose probit mortality data and resistance ratios for flupyradifurone**

**Table I in S1 Text. Log dose probit mortality data and resistance ratios for sulfoxaflor**

| **Strain** | **LC_50_**  **(mg/L)** | **95% CL** | **LC_95_**  **(mg/L)** | **95% CL** | **Resistance ratio** | |
| --- | --- | --- | --- | --- | --- | --- |
|  |  |  |  |  | **LC_50_** | **LC_95_** |
| Control | 59.4 | 56.1-62.8 | 90.6 | 83.9-99.9 | 1.0 | 1.0 |
| *α1^-/-^* | 54.8 | 50.0-60.3 | 85.3 | 75.3-103.2 | 0.9 | 0.9 |
| *α2^-/-^* | 82.0 | 73.9-91.0 | 142.7 | 122.8-181.6 | 1.4 | 1.6 |
| *α3^-/-^* | 207.8 | 185.6-236.3 | 306.0 | 263.2-402.5 | 3.5 | 3.4 |
| *α4^T227M^* | 73.0 | 50.8-108.0 | 166.2 | 110.7-594.5 | 1.2 | 1.8 |
| *α5^-/-^* | 55.7 | 50.7-61.3 | 88.5 | 77.9-107.6 | 0.9 | 1.0 |
| *α6^-/-^* | 63.0 | 57.3-69.0 | 96.6 | 86.0-115.7 | 1.1 | 1.1 |
| *α7^-/-^* | 56.6 | 51.5-62.1 | 88.2 | 78.0-106.2 | 1.0 | 1.0 |
| *β1^R81T^* | 948.5 | 726.2-1245.3 | 2280.2 | 1628.6-4643.7 | 16.0 | 25.2 |
| *β1^R81T^/+* | 116.3 | 102.9-131.0 | 175.1 | 152.0-222.1 | 2.0 | 1.9 |
| *β2^-/-^* | 50.3 | 46.4-54.8 | 76.2 | 67.6-91.7 | 0.8 | 0.8 |
| *β3^-/-^* | 64.2 | 59.7-68.1 | 89.7 | 83.9-98.4 | 1.1 | 1.0 |

| **Strain** | **LC_50_**  **(mg/L)** | **95% CL** | **LC_95_**  **(mg/L)** | **95% CL** | **Resistance ratio** | |
| --- | --- | --- | --- | --- | --- | --- |
|  |  |  |  |  | **LC_50_** | **LC_95_** |
| Control | 28.2 | 24.7-31.9 | 237.0 | 184.0-328.0 | 1.0 | 1.0 |
| *α1^-/-^* | 922.1 | 756.1-1066.0 | 12856.0 | 9343.9-19626.0 | 32.7 | 54.2 |
| *α1^+/-^* | 175.3 | 146.8-211.9 | 1923.8 | 1275.9-3377.0 | 6.2 | 8.1 |
| *α2^-/-^* | 484.4 | 266.6-985.3 | 13397.0 | 3076.9-71026.0 | 17.2 | 56.5 |
| *α2^+/-^* | 93.5 | 79.7-109.1 | 597.0 | 453.7-855.5 | 3.3 | 2.5 |
| *α3^-/-^* | 36.0 | 24.7-47.1 | 448.5 | 278.0-1005.9 | 1.3 | 1.9 |
| *α4^T227M^* | 18.2 | 13.8-22.8 | 73.4 | 51.4-141.1 | 0.6 | 0.3 |
| *α5^-/-^* | 18.0 | 1.0-15.2 | 43.3 | 34.2-63.5 | 0.6 | 0.2 |
| *α6^-/-^* | 28.7 | 24.3-34.0 | 142.5 | 105.2-219.3 | 1.0 | 0.6 |
| *α7^-/-^* | 30.0 | 24.5-37.0 | 238.1 | 157.0-440.0 | 1.1 | 1.0 |
| *β1^R81T^* | 4349.2 | 3096.0-6661.3 | 33601 | 17279.0-117310.0 | 154.0 | 141.8 |
| *β1^R81T/+^* | 71.2 | 57.8-87.5 | 686.4 | 459.6-1210.4 | 2.5 | 2.9 |
| *β2^-/-^* | 668.9 | 431.0-913.5 | 5797.4 | 3728.1-12256.0 | 23.7 | 24.5 |
| *β2^+/-^* | 56.2 | 46.7-66.7 | 309.4 | 234.2-453.3 | 2.0 | 1.3 |
| *β3^-/-^* | 28.2 | 24.7-31.9 | 237.0 | 184.0-327.9 | 1.5 | 0.7 |

**Table J in S1 Text. Log dose probit mortality data and resistance ratios for triflumezopyrim**

| **Strain** | **LC_50_**  **(mg/L)** | **95% CL** | **LC_95_**  **(mg/L)** | **95% CL** | **Resistance ratio** | |
| --- | --- | --- | --- | --- | --- | --- |
|  |  |  |  |  | **LC_50_** | **LC_95_** |
| Control | 1.3 | 1.2-1.4 | 2.5 | 2.2-3.1 | 1.0 | 1.0 |
| *α1^-/-^* | 1.8 | 1.2-2.6 | 4.0 | 2.7-13.5 | 1.4 | 1.6 |
| *α2^-/-^* | 0.8 | 0.7-0.9 | 1.8 | 1.5-2.3 | 0.6 | 0.7 |
| *α3^-/-^* | 1.2 | 1.0-1.3 | 2.6 | 2.2-3.5 | 0.9 | 1.0 |
| *α4^T227M^* | 1.8 | 1.3-2.5 | 4.4 | 3.0-11.1 | 1.4 | 1.8 |
| *α5^-/-^* | 1.4 | 1.2-1.6 | 4.0 | 3.2-5.6 | 1.1 | 1.6 |
| *α6^-/-^* | 55.6 | 45.8-68.1 | 134.3 | 101.8-218.1 | 42.8 | 53.7 |
| α6^+/-^ | 1.6 | 1.4-1.8 | 3.4 | 2.9-4.5 | 1.2 | 1.4 |
| *α7^-/-^* | 1.8 | 1.6-2.2 | 4.8 | 3.8-6.7 | 1.4 | 1.9 |
| *β1^R81T^* | 0.8 | 0.7-0.9 | 2.1 | 1.7-2.8 | 0.6 | 0.8 |
| *β2^-/-^* | 0.8 | 0.7-0.9 | 1.3 | 1.1-1.7 | 0.6 | 0.5 |
| *β3^-/-^* | 1.4 | 1.2-1.6 | 2.9 | 2.4-3.8 | 1.1 | 1.2 |
| *α5^-/-^*;*α7^-/-^* | 2.2 | 1.8-2.5 | 4.5 | 3.6-6.6 | 1.7 | 1.8 |

**Table K in S1 Text. Log dose probit mortality data and resistance ratios for spinetoram**

**Table L in S1 Text. Sequence identities between *Drosophila* nAChR subunits and corresponding orthologs in other insects**

|  | **Amel** | **Bter** | **Bmor** | **Mper** | **Tcas** |
| --- | --- | --- | --- | --- | --- |
| **α1** | 0.668 | 0.649 | 0.715 | 0.611 | 0.682 |
| **α2** | 0.74 | 0.741 | 0.779 | 0.654 | 0.743 |
| **α3** | 0.533 | 0.532 | 0.527 | 0.481 | 0.53 |
| **α4** | 0.761 | 0.5 | 0.712 | 0.685 | 0.726 |
| **α6** | 0.748 | 0.692 | 0.715 | 0.664 | 0.77 |
| **α7** | 0.609 | 0.608 | 0.779 | 0.625 | 0.747 |
| **β1** | 0.838 | 0.838 | 0.527 | 0.814 | 0.88 |
| **β2** | 0.698 | 0.695 | 0.712 | 0.595 | 0.691(*α*8)  0.717(*α*11) |

**Table M in S1 Text. Primers used in qPCR analysis**

| **Primer** | **Forward Sequences (5’-3’)** | **Reverse Sequences (5’-3’)** |
| --- | --- | --- |
| **Ribosomal protein L32** | GACGCTTCAAGGGACAGTATCTG | AAACGCGGTTCTGCATGAG |
| **nAChR α1** | TACGTTCGAGAAGCCCTACG | GGGAGCCTGCAGGATAATCA |
| **nAChR α2** | GGCCGCACGCAAAAAGTATC | CAAATGTGCCCACCAAGGATG |
| **nAChR α3** | CTGTCCGGAACTCCACAAGG | GTCGGAGCCTGCAGGATAAT |
| **nAChR α4** | CAGACGAAATAGCCGCCGTC | ACCACAACTGCCAACGTGA |
| **nAChR α5** | AGACAATGCCGGCTACTTCC | CAGCCAGCACAAAAACACGA |
| **nAChR α6** | GTGGAACGACTACAATCTGCG | AAGATACCAGGGGGCACGTA |
| **nAChR α7** | CCAATGTGCTCGATATAGACGATG | CTGTTATCCAACGCAGCTCCT |
| **nAChR β1** | GCAAATCCTGGCTGTTGTGC | ACGGTTAGCAGAGTTAACAGAGTT |
| **nAChR β2** | GCGTGACAGCATCAGCG | AGAGCCAGAGAAAGAAGCGG |
| **nAChR β3** | GGCCTGTTCACGAACTACGA | CAGACCTCGCTGGACTTCAA |

**Table N in S1 Text. The accession numbers of sequences used in Fig D in S1 Text.**

| Name | Protein ID |
| --- | --- |
| Dmelα1 | NP_001262916.1 |
| Dmelα2 | NP_524482.1 |
| Dmelα3 | NP_525079.3 |
| Dmelα4 | NP_001303394.1 |
| Dmelα5 | NP_001356885.1 |
| Dmelα6 | NP_995675.1 |
| Dmelα7 | NP_001285436.1 |
| Dmelβ1 | NP_523927.2 |
| Dmelβ2 | NP_524483.1 |
| Amelα1 | NP_001091690.1 |
| Amelα2 | NP_001011625.1 |
| Amelα3 | XP_016767553.1 |
| Amelα4 | XP_006562616.1 |
| Amelα5 | XP_392070.3 |
| Amelα6 | XP_026302163.1 |
| Amelα7 | XP_026300655.1 |
| Amelα8 | NP_001011575.1 |
| Amelβ1 | NP_001073028.1 |
| Bmorα1 | XP_021203288.2 |
| Bmorα2 | NP_001103397.1 |
| Bmorα3 | NP_001103387.2 |
| Bmorα4 | NP_001166816.1 |
| Bmorα5 | NP_001103392.1 |
| Bmorα6 | XP_037866711.1 |
| Bmorα7 | XP_037866711.1 |
| Bmorα8 | XP_037874245.1 |
| Bmorβ1 | NP_001166819.1 |
| Bterα1 | XP_003397561.2 |
| Bterα2 | XP_003397559.1 |
| Bterα3 | XP_003399573.1 |
| Bterα4 | XP_003398630.2 |
| Bterα5 | XP_020722434.1 |
| Bterα6 | XP_020723485.1 |
| Bterα7 | XP_012167932.1 |
| Bterα8 | XP_012163745.1 |
| Bterβ1 | XP_003393394.1 |
| Mperα1 | XP_022166094.1 |
| Mperα2 | CAA57476.1 |
| Mperα3 | CAB52297.1 |
| Mperα4 | CAI54101.1 |
| Mperα6 | CAI54103.1 |
| Mperα7 | XP_022170816.1 |
| Mperα8 | XP_022167599.1 |
| Mperβ1 | XP_022165274.1 |
| Tcaα1 | EEZ99265.1 |
| Tcaα2 | EFA10793.1 |
| Tcaα3 | NP_001107770.1 |
| Tcaα4 | EEZ99194.2 |
| Tcaα5 | EFA02896.1 |
| Tcaα6 | KYB29613.1 |
| Tcaα7 | EFA12057.1 |
| Tcaα8 | ABS86912.1 |
| Tcaα11 | ABS86915.1 |
| Tcaβ1 | EFA12056.2 |
