# Supplementary material for: Nicotinic acetylcholine receptor modulator insecticides act on diverse receptor subtypes with distinct subunit compositions
Source: PLoS Genet. 2022 Jan 19;18(1):e1009920. doi: 10.1371/journal.pgen.1009920 (PMC8803171; doi:10.1371/journal.pgen.1009920)
Supplement: Attachment — Submitted filename: PG response Letter.docx [file pgen.1009920.s003.docx]

Dear Dr. Palli,

Thank you for your letter and for the reviewers’ comments concerning our manuscript entitled “Nicotinic modulation insecticides act on diverse receptor subtypes with distinct subunit compositions” (PGENETICS-D-21-01445). Those comments are valuable and very helpful for revising and improving our paper. We studied comments carefully and revise the manuscript which we hope meet with approval. Revised parts are marked in the paper. Here are our point-by-point responses:

Yours sincerely,

Jia Huang

Professor, Ph.D

Institute of Insect Sciences

Zhejiang University

Hangzhou, 310058, China

Reviewer #1: This manuscript describes the use of 10 mutant lines of Drosophila to investigate the role of different nAChR subunit subtypes in mediating the response to insecticidal agonists of the nAChR. While the work performed in the study is conceptually very simple, the results obtained are interesting and provide new information on this topic. However, many overblown and misleading claims are made in the manuscript that are not supported by the data, these significantly diminish the quality of the presented paper. The manuscript also suffers from several issues in regards its presentation and description of the work done.

Major comments

The quality of the written English in the manuscript is poor in places and requires revision prior to publication.

Response: We sent the manuscript to AJE for English editing service and it is now polished by native speakers.

In the title, abstract and elsewhere, the authors use the term: ‘nicotinic modulation insecticides’ this is at best ambiguous and unclear. Please avoid use of this term and replace with a suitable alternative, such as ‘insecticides acting on the nicotinic acetylcholine receptor’.

Response: Thank you very much for your suggestion and we replaced this term in the title, abstract and main text.

Line 47/48. The authors state: ‘Although all act via insect nicotinic acetylcholine receptors, the mode of action is unclear.’ This is misleading, actually a great deal of work has been done on the MOA of these insecticides and their action at the nAChR. Thus this claim should be removed or modified.

Response: Thank you very much for your advice and we revised it to “which is not fully understood at the molecular level”

Line 80: The authors state: ‘Thus, neonicotinoids account for 24% of the global insecticide’, however at line 44 they state: ‘The neonicotinoids and spinosyns make up about 27% of the insecticides by world market value’. This discrepancy should be corrected.

Response: We revised it to “Neonicotinoids and spinosyns account for approximately 24% and 3% of the world market value of insecticides, respectively”.

Line 127/128: The authors state that knock out of two of 10 nAChR subunits (α4 and β1) was homozygous lethal, however they then start talking about the introduction of two mutations into these subunits. The link here is unclear. Furthermore why not use one of the many genetic tools available in Drosophila (i.e. balancers etc.) to investigate these subunits in the heterozygous form? If this is actually what they did please make this significantly more obvious (and how this was achieved in the results).

Response: Thank you very much for your questions. When an insecticide acts by modulating an ion channel like nAChR, the dysfunction of a few channels is usually sufficient to cause death. Thus, the heterozygous KO insects which possess 50% of sensitive channels are still similar to susceptible insects in the presence of insecticide. We also tested the heterozygous KO flies in bioassays (like αx^+/-^ in Table S1-S11), most of them showed no or low resistance when their homozygous KO mutants (αx^-/-^) were resistant to insecticides. Therefore, we used point mutation alleles of α4 and β1 in all experiments instead of heterozygous KO flies. We added more explanations in the revised text (line 133-137).

Lines 219: The authors state: ‘However, our results clearly showed that sulfoxaflor and flupyradifurone may specifically act on the same nAChR subtype which consists of α3 and β1 subunits’. I don’t see how the authors can make this claim based on the work performed. Evidence is presented to suggest that nAChRs containing these subunits are sensitive to these insecticides, but conclusions on which other nAChR subunit types co-assemble with them cannot be established based on the work done. What the data does, and does, not show needs to be much more clearly and carefully defined.

Response: Thank you very much for your suggestion. We revised this sentence and in line308-312, we emphasized again that there could be genetic redundancy, especially between some α-type subunits which may form other receptor subtypes as secondary targets of tested insecticides.

Lines 248-252: The authors state: ‘Such unusual phenomenon can be partially explained by our findings that the seven neonicotinoids have at least three distinct molecular targets in vivo. To some extent, the continuous use of different neonicotinoids is a kind of spontaneous insecticides rotations, which has been proven to be effective in mitigating or delaying resistance’. This is a very bold claim that I believe to be misleading, inaccurate and not supported by phenotypic investigation of the impact of the known resistance mutations. Specifically, R81T has been shown to confer resistance to multiple neonicotinoid insecticides, and this finding directly contradicts this conclusion.

Response: Thank you very much for your suggestion and we removed this claim. Only the β1^R81T^ flies caused high resistance to all neonicotinoids, that may explain why R81T is the only target-site resistance found in the field.

Line 277: The authors claim: ‘The α6 subunit has been proposed as the main target of spinosyns since the resistance to spinosad in many insects is associated with loss-of-function mutations in the α6 gene [24], however, whether other subunits are involved is still unknown.’ Again this is a somewhat misleading statement that discredits work done by numerous labs on this topic. There is unequivocal evidence that the α6 subunit IS the main target of spinosyns and absolutely no evidence at all that any other subunits are involved. Thus the work presented on this topic by the current authors is not novel and any claims that it is are inaccurate and should be moderated.

Response: Thank you very much for your suggestion and we revised this part. We know that the α6 subunit has been proposed as the main target of spinosyns since the field evolved resistance to spinosad is associated with loss-of-function mutations of α6 loci in many pest insects [25-32]. However, the involvement of other subunits, such as α5 and α7, which are phylogenetically close to α6, has not been clarified (S4 Fig). Previous reports showed that α5 and α7 can form functional homomeric and heteromeric channels in vitro while α6 can only form heteromeric channels with α5 or α5/α7 together [33, 34]. We then wondered whether there was genetic redundancy among these evolutionarily conserved subunits. For the first time we found that deletion of α5 and α7, alone or in combination, did not lead to resistance, confirming that spinosyns may exclusively act on the α6 homomeric nAChR.

Lines 312: The authors state: ‘Since most Drosophila nAChR subunit genes (except α5 and β3) have one-to-one orthologs in the honeybee and bumblebee genomes [7], the expression and assembly of receptors could be conserved between flies and bees, suggesting that our results will enable further studies about the ecotoxicology and risk assessment for these nAChR modulators.’ This is a very big jump, just because two different insect species have orthologous genes does not mean that inferences on one can be applied to another, especially when the two species are separated by millions of years of evolution and have completely different life histories!

Response: Drosophila genetics has been a powerful approach to identify molecular targets for insecticides (e.g. Met for Methoprene; Rdl for cyclodienes and so on, for review see Scott and Buchon, 2019, *Pestic Biochem Physiol*). Recently, other labs and our lab also employed Drosophila reverse genetics and behavioral assays to identify the molecular targets for pymetrozine (Nesterov et al, 2015, *Neuron*) and amitraz (Guo et al., 2021, *eLife*), respectively. Actually, the MOA of insecticides is conserved between Drosophila and other insects, probably because insecticides disrupt essential physiological functions. On the other hand, previous reports showed that the nAChR family are evolving slowly [47] and the core groups of nAChR subunits are highly conserved among different insects spanning ~300 million years of evolution (S4 Fig), probably due to their essential roles in the nervous system. For some subunit genes, even the alternative splicing and RNA editing are conserved (Jones et al., 2006, *Genome Res*; Jin et al., 2007, *BMC Evol Biol*). We also added a supplementary table (S12) to show that the sequence identities between orthologs are very high. This part was revised accordingly in line 324-330.

Reviewer #2: Lu et al. investigated the impact and toxicity of modulators (IRAC MoA group 4) of the nicotinic acetylcholine receptor (nAChR) in transgenic flies either lacking individual receptor subtypes, or – in two cases – flies with mutated subunits, because their knock-out was lethal. In total the authors investigated 11 insecticides including neonicotinoids, butenolides, sulfoximines, mesoionics and spinosyns. They convincingly demonstrated by RT-qPCR that the different mutant fly lines lack the respective subunits they are supposed to lack. The authors conducted bioassays with each transgenic line in comparison to wildtype flies and calculated resistance ratios for each insecticide. Furthermore, the authors conducted climbing assays with each fly line and mimicked the symptomology of poisoning for some insecticides by thermogenetic activation of nAChR expressing neurons. Finally, they conducted fecundity and development assays with flies expressing a mutant R81T ß1 subunit, which is known to confer neonicotinoid resistance in aphids.

The study is well executed and the methods are described in enough detail. The introduction and discussion are well referenced and there is not much to criticize. Many of the presented findings are novel and advance the knowledge in the field of insect nAChR modulator mode of action. However, employing Drosophila as a proxy may not always reflect the same effects one would expect in pest insects or beneficials targeted by these insecticides, especially since dipterans and hymenopterans diverged approx. 300 mya. However, as the authors outlined, in many sequenced insect genomes 1:1 orthologs for the different nAChR subunits are present, thus justifying some of the claims made by the authors. It is of particular interest that the ß1 subunit seems most crucial for all insecticides tested, except spinetoram. I have only a few minor points I want the authors to address and listed below.

1) I would suggest to slightly change the title as “nicotinic modulation insecticides” sounds a bit awkward. I think “Nicotinic acetylcholine receptor modulator insecticides act on…” would be better.

Response: Thank you very much for your suggestion and we changed the title.

2) L25: add “bee” pollinators

Response: added.

3) L27: I do not think that the MoA of these insecticides is unclear for 30 years. Please reword, because many good papers were published on the molecular MoA of these insecticides.

Response: Thank you very much for your advice and we revised it to “which is not fully understood at the molecular level”

4) L69: I do not agree that for MANY insecticides the exact molecular targets remain elusive. Please tone down.

Response: Thank you very much and we changed it to “some”.

5) L245: Imidacloprid was the first neonicotinoid launched to the market in 1991, so these insecticides are around for three decades, not four.

Response: corrected.

6) L351: Insecticide bioassay – how many replicated were tested?

Response: added in the methods.

7) L402: Climbing assay – how many replicated were tested?

Response: added in the methods.
